# Nanofluid impingement jet heat transfer

**DOI:** 10.1186/1556-276X-7-139

**Published:** 2012-02-17

**Authors:** Obida Zeitoun, Mohamed Ali

**Affiliations:** 1 King Saud University, Mechanical Engineering Department, King Abdullah Institute for Nanotechnology, 800 Riyadh, 11421, Saudi Arabia

**Keywords:** nanofluid, heat transfer, impingement jet

## Abstract

Experimental investigation to study the heat transfer between a vertical round alumina-water nanofluid jet and a horizontal circular round surface is carried out. Different jet flow rates, jet nozzle diameters, various circular disk diameters and three nanoparticles concentrations (0, 6.6 and 10%, respectively) are used. The experimental results indicate that using nanofluid as a heat transfer carrier can enhance the heat transfer process. For the same Reynolds number, the experimental data show an increase in the Nusselt numbers as the nanoparticle concentration increases. Size of heating disk diameters shows reverse effect on heat transfer. It is also found that presenting the data in terms of Reynolds number at impingement jet diameter can take into account on both effects of jet heights and nozzle diameter. Presenting the data in terms of Peclet numbers, at fixed impingement nozzle diameter, makes the data less sensitive to the percentage change of the nanoparticle concentrations. Finally, general heat transfer correlation is obtained verses Peclet numbers using nanoparticle concentrations and the nozzle diameter ratio as parameters.

## Introduction

Fluid heating and cooling play very important roles in many industries including power generation, production processes, transportation and electronics. Heat transfer can be enhanced using different methods such as extended surfaces (fins), vibration of the heated surfaces, injection or suction of the fluid and applying electrical or magnetic fields. Nanofluid heat transfer is an innovative technology which can be used to enhance the heat transfer. The term nanofluid refers to a new kind of fluid produced by suspending nanoparticles in the base fluid.

Impinging liquid jet is an established technique to provide high local heat transfer coefficients between the impinged liquid and a surface. This cooling technique is considered as an attractive cost effective method of cooling [1,2]. Combining the liquid jet impingement and the nanofluid technologies is thought to capture the advantages of both and consequently enhances the heat transfer significantly. Enhancing the heat transfer means compact size and low weight which reduces the cooling system capital cost.

Liquid jets can be classified as submerged or free surface. A submerged jet is formed when a liquid jet is discharged into the same liquid medium. A free surface jet is formed when a liquid jet is discharged into a gas medium. For free surface jet, the liquid jet impingement has demonstrated high cooling capacity as reported by Liu and Lienhard [1], Lienhard and Hadeler [2], and Bergles [3]. The Jet impingement or free surface flow can be classified according to jet orientation, surface type or flow type as vertical or horizontal, flat or curved, and single or two phase flow, respectively.

Upon liquid jet impinging on a horizontal plate, the liquid spread due to inertia and gravity forces. A hydraulic jump occurs when the flow suddenly changes from shooting flow, before the hydraulic jump, to streaming flow after the hydraulic jump. This jump is accompanied by a sudden increase in the liquid film thickness. This change is accompanied by a significant decrease in the heat transfer. Jambunathan et al. [4] reviewed previous works of heat transfer between circular liquid jet and horizontal surface. Review of previous investigations reveals very high heat transfer rates at the stagnation point of the jet due to the very thin boundary layer at the stagnation point. However, at a distance of two to three nozzle diameters from the stagnation point, the cooling rate is less than half that of the stagnation value.

The pioneer work of Watson [5] has divided the flow field of an impinging jet over a horizontal circular disk before the hydraulic jump into two regions. The first region is near the center of the jet, where he assumed a boundary layer type flow. The second part was regarded as a free surface flow up until the hydraulic jump position. The region before the hydraulic jump is characterized by a high heat transfer capability. Chaudhury [6] followed Watson [5] and solved the energy equation, based on integral analysis, taking into consideration the viscous dissipation. The thermal boundary condition at the circular plate was assumed to be of the constant wall temperature type.

Experimental investigations of impingement jet heat transfer include works of Ishigai, Zhao and Masuoka, Baonga et al. and Teamah and Farahat [7-10]. Ishigai. [7] studied experimentally the flow and heat transfer of an impinging round jet over a horizontal plate. They compared their experimental measurements of the film thickness with the theoretical predictions of Watson [5]. There was a good agreement between the two solutions near the center of the jet, but as the radial location increases, the difference becomes wider. Zhao and Masuoka [8] have investigated flow and heat transfer due to liquid jet impingement on a circular surface. They have studied the heat transfer between small jets of 0.9 and 2 mm and a disk of 10 mm diameter. Baonga et al. [9] investigated liquid film, hydraulic jump and local heat transfer distributions along the radial direction of a circular disk. For jet Reynolds number in the range of 1,050 to 9,000, and for each nozzle diameter, the difference between the stagnation and average Nusselt numbers decreases significantly for higher Reynolds number. When the jet Reynolds number increases, the average heat transfer coefficient increases because of the increase in the liquid flow rate. Furthermore, Teamah and Farahat [10] have investigated both the heat transfer and fluid flow due to the impingement of vertical circular water jet on a horizontal heated surface numerically and experimentally. However, the hot surface used in their experiment was square of 0.95 m side.

There are few models available for the average liquid jet impingement Nusselt number along circular disks; such models are those given by Zhao et al. [8] and Teamah and Farahat [10]. Integral analysis method was used by [8] to solve the flow along the radial direction of the circular disk. The film along the radial direction was divided into stagnation, near impingement point, boundary layer and similarity regions. Based on integral analysis results, Zhao et al. [8] have developed the following model:

(1) $$Nu_{j}=\text{0}\text{.77212}{\left(\frac{\text{2}Dj}{D}\right)}^{\text{2}}Pr^{\text{0}\text{.4}}Re_{j}^{\text{0}\text{.5}}+\text{0}\text{.89}{\left(\frac{\text{2}D_{j}}{D}\right)}^{\text{2}}\left[{\left(\frac{D}{\text{2}D_{j}}\right)}^{\text{3}/\text{2}}-\text{1}\right]Pr^{\text{1}/\text{3}}Re_{j}^{\text{0}\text{.5}}$$

where *Nu_j _*is the Nusselt number based on the jet nozzle diameter *D_j_, D *is the disk diameter; *Pr *is the Prandtl number and *Re_j _*is the Reynolds number based on jet nozzle diameter. For the complete list of the symbols used in all equations, please refer to the 'Appendix' section. Teamah and Farahat [10] developed a mathematical two-dimensional model in radial and vertical directions. They solved the governing equations numerically up to *r */*D_j _*= 50 in the radial direction. However, they integrated heat transfer results in steps along the radial direction, from *r *= 0 into *r *to obtain average Nusselt number as a function of the radial positions. Based on their numerical data, they have obtained two correlations based on jet Reynolds number. For *Re_j _*range from 5,000 to 20,000:

(2) $$Nu_{j}=\text{5}\text{.693}{\left(\frac{D}{\text{2}D_{j}}\right)}^{-\text{1}\text{.508}}Re_{j}^{\text{0}\text{.56188}}$$

Different jet orientations were also investigated by Silverman and Nagler, Tong and Rahman et al. [11-13]. Silverman and Nagler [11] investigated heat transfer between a horizontal water jet and a vertical surface. Tong [12] investigated the effect of liquid inclination angle on the hydrodynamics and heat transfer of the impingement of a liquid jet on a horizontal surface. The locations of the maximum Nusselt number as well as maximum pressure on the surface were found to be identical with the geometric jet impingement point. Rahman et al. [13] solved free surface flow in the presence or absence of gravity. The distribution of film height was found to be strongly affected by the magnitude and orientation of gravity.

Liquid properties effect on free surface flow was investigated by Sun et al. and Liu et al. [14,15]. Sun et al. [14] carried out local measurements to investigate the characteristics of heat transfer from small heater to liquid jet of Prandtl number between 7 and 262. The Nusselt number dependence of *Pr*^1/3 ^was testified by their experimental data, as well as the data of water and heavy electrochemical liquid from other resources. Bula et al. [16] investigated effect of high Prandtl number fluid for jet impinging perpendicularly on a solid substrate of finite thickness containing small discrete heat sources. It was found that the local heat transfer coefficient had maximum value at the center of the disk and decreases gradually with radius as the flow moves downstream. Liu et al. [15] focused on heat transfer at the stagnation point considering in their analysis the effect of surface tension.

Carper et al. [17] investigated experimentally the heat transfer for impinging liquid jet on a rotating disk. They used petroleum oil as the working medium. The heat transfer coefficients increase approximately with the square root of the rotational velocity of the disk. For two phase flow applications, Robidou et al. [18] investigated the boiling on a hot plate cooled by a water jet. In the forced convection regime, they found that heat fluxes increased with the increase of the subcooling, jet velocity and decrease of the distance from the stagnation line. Boiling first starts in the parallel flow region and propagates in the direction of the jet. In the fully developed nucleate boiling regime, no influence of jet velocity, subcooling and the nozzle-to-plate spacing on the heat flux in the parallel flow region were observed.

Liquid jet and spray impingement cooling were studied experimentally by Oliphant et al. [19]. The comparison of the two cooling techniques revealed that spray cooling can provide the same heat transfer coefficient as jets at a substantially lower mass flux. It was concluded that the effective cooling of non-boiling sprays was primarily due to the unsteady boundary layer resulting from droplet impact and secondarily from evaporative cooling. For nanofluids, considerable works are concentrated on the thermal property measurements and modeling including the study of Williams et al. [20]. Experimental investigations have revealed that nanofluids have remarkably higher thermal conductivities.

Comprehensive reviews of nanofluid heat transfer have been presented by Trisaksri and Somchai [21] and Wang and Mujumdar [22]. Trisaksri and Somchai [21] have concluded that, among a lot of models for thermal conductivity it is not clear yet which model can be considered as the best. They also added that the suspended nanoparticles remarkably increased the forced convective heat transfer of the base fluid. In addition to that, they also noted that the heat transfer of the nanofluid increased as the volume fraction increased for fixed Reynolds number. Wang and Mujumdar [22] reported that the use of nanofluids in a wide range of applications appears promising, but the development of this field faces several challenges: (1) the lack of agreement between experimental results from different groups, (2) the often poor performance of suspensions (3) and lack of the theoretical understanding of the mechanisms. Further theoretical and experimental research investigations were needed to understand the heat transfer characteristics of nanofluids.

For nanofluid flow inside circular tube, experimental and numerical results have indicated that increasing the nanoparticle concentration enhanced the heat transfer coefficient on the cost of the pressure drop [20,23] and [24]. It has been found by Torii [23] that significant enhancement of heat transfer performance was observed in comparison with pure water. This enhancement was intensified with the increase in the Reynolds number and the nanoparticle concentration.

Magïa et al. [25,26], Nguyen et al. [27-29] and Gherasim et al. [30] carried out experimental investigations to study the heat transfer performance of nanofluid (Al_2_O_3_) for confined and submerged impinging jets. Experimental data, obtained for turbulent flow regime, have clearly shown that the inclusion of nanoparticles into distilled water has produced a considerable enhancement of the convective heat transfer coefficient. For a particular nanofluid with 6.8% particle volume concentration, the heat transfer coefficient has been found to increase as much as 40% compared to that of the base fluid. Data of Nguyen et al. [28] indicate that the use of a nanofluid can provide a heat transfer enhancement. It has been observed that the highest surface heat transfer coefficients can be achieved using an intermediate nozzle-to-surface distance of 5 mm and a 2.8% nanoparticle volume fraction. Nanofluids with 6% or higher particle volume fraction have been found not appropriate for the heat transfer enhancement purpose under the confined impinging jet configuration.

Manca et al. [31] and Feng and Kleinstreuer [32] numerically investigated the coffined nanofluid jet heat transfer. The numerical data of Manca et al. [31] indicate that Nusselt number increases for increasing particle concentrations and Reynolds numbers. A maximum increase of 18% is detected at a concentration of 6%. The required pumping power as well as Reynolds number increase as the particle concentration grows, which is almost 4.8 times greater than the values calculated for the case of base fluid.

For nanofluid jet boiling, Chakraborty et al. [33] have investigated the cooling of hot steel plate using TiO_2 _nanofluid jet. Their results of water based-TiO_2 _nanofluid showed significantly higher cooling rate as compared to the water as a coolant. Chakraborty et al. [33] concluded that convective heat transfer by jet boiling of the nanofluid together with the lower surface tension and higher viscosity of the nanofluid could be important factors leading to this faster cooling.

Nanofluid spray boiling is investigated by Chang et al. [34] and Bellerová et al. [35]. Chang et al. [34] found that the optimal heat transfer performance is obtained using Al_2_O_3 _particle volume fraction of 0.001%. In spray cooling with high-volume-fraction nanofluids (0.025 to 0.05%), they found that the nanoparticles were easily deposited on the heated surface, thereby, reducing the number of active nucleation sites and hindering the convection heat transfer mechanism between the surface and the nanofluid. Low-volume-fraction nanofluids (0.001%) yield a significant improvement in the spray cooling efficiency since most of the nanoparticles rebound from the heated surface directly or are washed away by subsequently arriving droplets. The results of Bellerová et al. [35] have found, by comparing the nanofluid results with that of pure water, that an approximately 45% decrease of heat transfer coefficient of spray cooling with the volume fraction of the nanoparticle suspension increasing from 0 to 16.45%. The reduction of heat transfer coefficient caused by the change of the spraying impact duration due to the presence of nanoparticles.

This paper shows the effect of using a vertical alumina-water nanofluid jet with different concentrations on the cooling of horizontal heated circular disks with different diameters in terms of dimensionless parameters. This paper is focused on the effect of disk circular size and its relation to the nanofluid concentration on the cooling process.

## Experimental details

**Figure 1:**
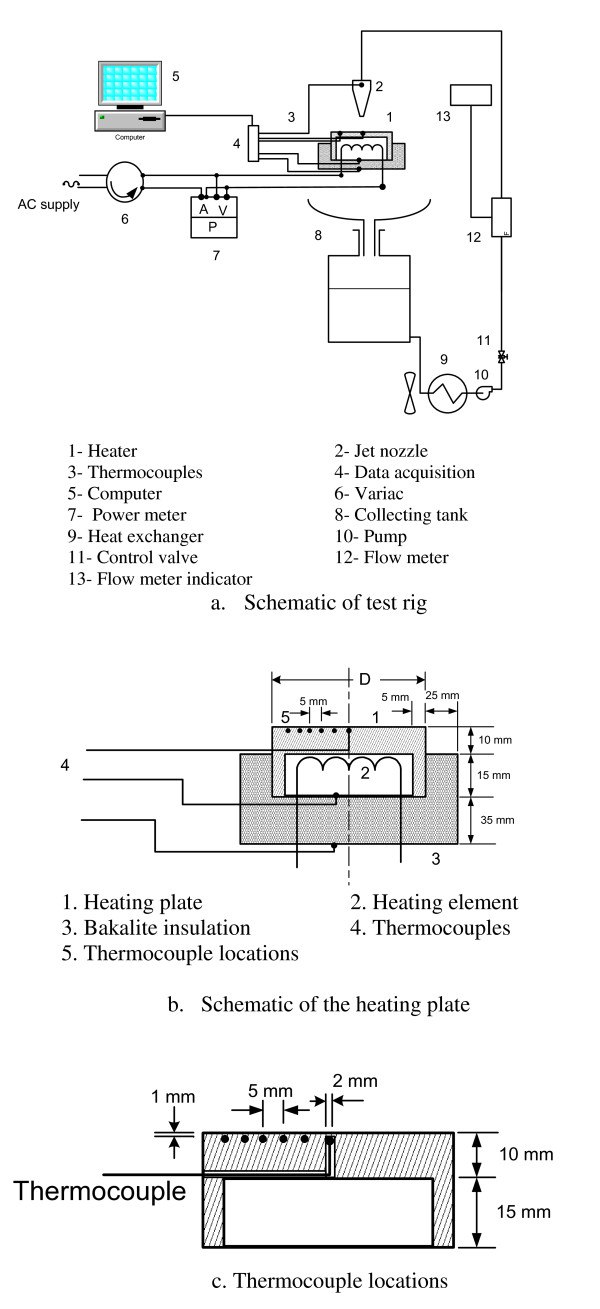
**Experimental setup**. (**a**) Schematics of test rig and (**b**) heating plate and (**c**) thermocouple locations.

The experimental test rig of the current experiment consists of a liquid (water or nanofluid) circuit and a hot circular disk as shown in Figure 1a. Water (or nanofluid) flows from the collecting tank (8) through a heat exchanger (9) using a pump (10). The flow rate is controlled and measured by a valve (11) and a turbine flow meter (12), respectively. The water jet emerges from the nozzle and impinges on a horizontal hot circular disk (1). The falling liquid is collected in a collecting tank (8). A heat exchanger (9) with fan is used to cool the fluid. The circular disk is heated using an electric heater as shown in Figure 1b. The power of the heaters is controlled using a variac transformer (6) (Slideup type made by Voltac Co. Ltd, Tokyo, Japan). The voltage across the heater, the current through the heater and the electric power are measured by a power meter (7) (Metrix PX110; Metrix Instruments, Paris, France). The uncertainities in volt, current and power are ± 0.5, ± 0.7 and ± 1.5 of readings, respectively. The temperatures of the hot disk (1), the jet and the bakalite insulation are measured using k-type thermocouples. The thermocouples (3) are connected to a data acquisition system (4) where the data is collected and stored on a labtop computer (5) for further analyses. The data acquisition type is OMB-DAQ-56 made by Omega Engineering, Inc. (Stamford, CT, USA).

Three nozzles of different diameters are used in the current investigation 0.0039, 0.0055 and 0.0082 m. The nozzle length to diameter ratio is kept above 20 to avoid the effect of entrance on jet exit. The height of the nozzle above the hot disk is maintained constant at 50 mm during this investigation. In addition to that, four circular heating plates are used with diameters 0.080, 0.100, 0.115 and 0.133 m. The first two are made of copper and the others of aluminum, respectively. The disks were nickel-electroplated to avoid rusting. A Bakelite insulating housing (3) is built to enclose the circular disk as shown in Figure 1b. The thickness of the hot disks is 25 mm. The electric heaters used are spiral stainless steel heating elements of 0.008-m diameter. The heater is placed in a circular gap of 0.015-m height as shown in Figure 1b. High thermal conductivity cement is used to hold the heater in its cylindrical gap housing. K-type thermocouples made by Thermocoax (Suresnes Cedex, France) are used to measure the disk surface temperatures. The thermocouples are of insulated junction and sheathed by stainless steel of 0.0005-m outside diameter. Those thermocouples are located along the radial direction of the disk each 0.005 m apart as shown in Figure 1c. The thermocouples are impeded in 0.002-m diameter holes in the disk using Omega high thermal conductivity cement (Omega Engineering, Inc., Stamford, CT, USA). The holes are 0.001 m below the disk surface. Two more thermocouples are used to measure the temperature difference across the Bakelite insulation. Water jet temperature is measured using a thermocouple before jet exit.

**Figure 2:**
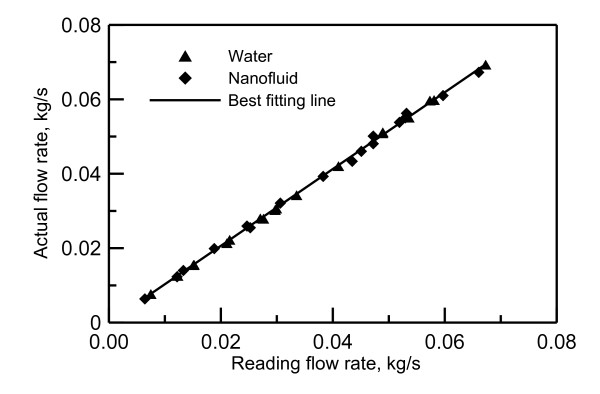
**Flow meter calibration**.

The thermocupoles and data acquisition system are calibrated at ice and boiling points. At the ice point, the error in thermocople readings are in the range from -0.2 to 0.1°C. At the boiling point, the error in the thermocople readings are in the range from 0.15 to 0.4°C. The flow meter used is a turbine flow meter (FTB602) made by Omega Engineering, Inc. (Stamford, CT, USA). The flow meter is calibrated using a balance scale and a stop watch. The calibration data for water and nanofluid are shown in Figure 2. The difference between the reading of the flow meter and that of the measured flow rates is -2% at most.

## Nanofluid preparation

Alumina nanofluid (10-nm particles of Al_2_O_3 _dispersed in water) of 20% mass concentration supplied by the Nanostructured & Amorphous Materials, Inc. (Houston, TX, USA) is used in the present investigation. The manufacturer information about this nanofluid is that the average nanoparticle size = 10 nm gamma and it looks as a transparent liquid. The manufacturer 20%-mass concentration nanofluid was diluted to different concentrations using distilled water. These new diluted solutions are ultrasonic vibrated for about 6 h to insure complete dispersions of the nanoparticles. Still camera is used to check any precipitation. Photographs are taken just after ultrasonic vibration and 48 h later. No precipitation is observed during this period. Two mass concentrations, 6.6% and 10%, are used in the current investigation.

**Figure 3:**
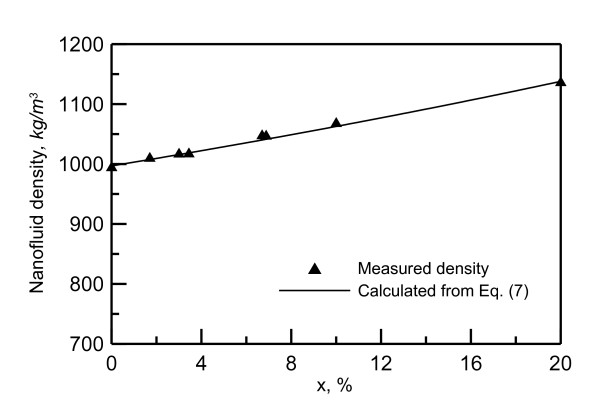
**Nanofluid density at ambient temperature of 25.5°C**.

**Figure 4:**
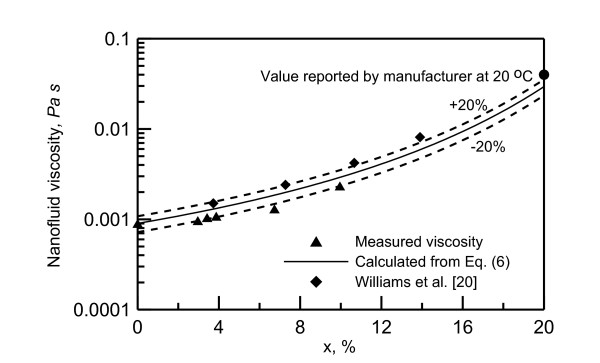
**Nanofluid viscosity at 24.5°C**.

**Figure 5:**
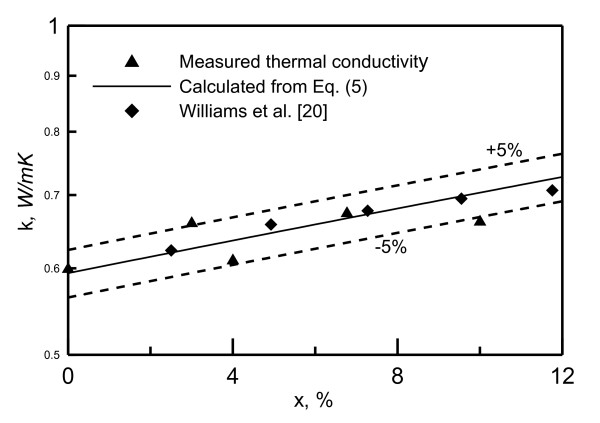
**Nanofluid thermal conductivity at 26.5°C**.

Nanofluid density is measured using DMA 35N density meter. Figure 3 shows the effect of mass concentration on the measured nanofluid density at ambient temperature of 25.5°C. The nanofluid viscosity at ambient temperature 24.5°C is measured using Viscolite 700. The Effect of nanoparticle concentration on viscosity is shown in Figure 4. As seen in this figure, the viscosity value is doubled as the concentration increases from 0% to 10%. The nanofluid thermal conductivity is measured using KD2Pro. The Effect of of nanoparticle concentration on thermal conductivity is shown in Figure 5. As shown in this figure, the thermal conductivity increased about 15% as the concentration increases from 0% to 10%.

Comparison with the viscosity and the thermal conductivity models available in the literature revealed that the models proposed by Williams et al. [20], represented by Equations 3 to 6, can reasonably present the current measurements:

(3) $$\rho =\varphi \rho_{\text{p}}+\left(1-\varphi \right)\rho_{\text{b}}$$

(4) $$C=\frac{\varphi \rho_{\text{p}}C_{\text{p}}+\left(1-\varphi \right)\rho_{\text{b}}C_{\text{b}}}{\rho }$$

(5) $$k=k_{\text{p}}\left(1+4.5503\varphi \right)$$

(6) $$\mu =\mu_{\text{b}}\exp\left[4.91\varphi /\left(0.2092-\varphi \right)\right]$$

where *φ *is the nanoparticle volumetric concentration, *ρ, C, k *and *μ *are the nanofluid density, specific heat, thermal conductivity and viscosity, respectively. For the based fluid, *ρ*_b_, *C*_b_, *k*_b _and *μ*_b _are the density, specific heat, thermal conductivity and viscosity, respectively. For the nanoparticles, *ρ*_p_, *C*_p _and *k*_p _are the density, specific heat and thermal conductivity, respectively. The relation between mass concentration × and the volumetric concentration *φ *can be estimated from the following equation and Equation 3:

(7) $$1/\rho =X/\rho_{\text{p}}+\left(1-X\right)/\rho_{\text{b}}$$

where *X *is the nanofluid particle mass concentration; *ρ*_p_, fluid density of a nanoparticle; *ρ*_b_, fluid density base. The measurred viscosity falls within ± 20% of Equation 6, and the measurred thermal conductivity falls within ± 5% of Equation 5.

## Experimental procedure

Experiments are carried out to examine the effect of nanofluid flow rates and concentrations on steady state cooling of horizontal circular disks. Results are obtained for mass concentrations of 0.0%, 6.6% and 10.0%, mass flow rate in the range 0.006 to 0.075 kg/s and for heat flux in the range 60 to 100 kW/m^2^. Water is used as liquid reference (0 concentrations) in the current investigation since it is the base fluid of the used nanofluid. Three nozzles are used to examine jet size effect on heat transfer from hot disks. Four disks are used to examine effect of disk size on heat transfer.

At the beginning of the experiment, the control valve is used to establish the required flow rate through the nozzle. Then, the heater is turned on where the electric power is adjusted using the variac transformer and recorded. The data acquisition starts to collect thermocouple readings and saves them in the computer. It should be noted that the system reaches steady state in 30 min where the temperatures are recorded through the data acquisition system. The experiments are done first for pure water only, then for nanofluid with different concentrations.

## Data analysis

The average temperature *T*_wam _of the hot surface is calculated as:

(8) $$T_{\text{wam}}=\frac{\overset{8}{\underset{i=1}{\sum }}T_{\text{wk}}A_{\text{k}}}{A}$$

where *T*_wk _is the local wall temperature for the element area *A*_k_. *T*_wam _is the average temperature of the local wall. The heat loss *Q*_loss _through the Bakelite housing is estimated as:

(9) $$Q_{\text{loss}}=\frac{k_{\text{ins}}A_{\text{ins}}}{\delta_{\text{ins}}}\Delta T_{\text{ins}}$$

where *k*_ins _is the insulation thermal conductivity, *A*_ins _is the insulation disk area, Δ*T*_ins _is the temperature difference across the insulation and *δ*_ins _is the insulation thickness. The heat transfer rate Q between the jet and the hot surface is calculated from the following equation:

(10) $$Q=Q_{\text{ele}}-Q_{\text{loss}}$$

where *Q *is heat transfer and *Q*_ele_, electrical heat transfer. The average temperature *T*_wam _of the hot surface is obtained by correcting *T*_wam _due to the effect of metal thickness, *δ_th _*= 0.001 m between the hot surface and the thermocouple locations as seen in Figure 1c:

(11) $$T_{\text{wa}}=T_{\text{wam}}-\frac{{\delta_{th}}^{}q}{k_{\text{m}}}$$

where *k*_m _is the local temperature of metal and *q *is heat flux. The average heat transfer coefficient based on temperature difference between the wall average temperature *T*_wa _and the water jet temperature *T*_j _(before striking the hot surface) can be calculated as:

(12) $$h=\frac{q}{\left(T_{\text{wa}}-T_{\text{j}}\right)}$$

where *h *is the average heat transfer coefficient. The Nusselt number, Nu*_D _*based on disk diameter is given by:

(13) $$Nu_{D}=\frac{hD}{k}$$

where *hD *is the average heat transfer coefficient of the disk diameter. The jet velocity at the nozzle exit, V_j_, is obtained using the continuity equation at the nozzle exit. The jet velocity at impingement point can be obtained by applying the energy equation between the nozzle exit and just before the impingement point as:

(14) $$V_{i}=\sqrt{V_{j}^{2}+2gZ_{o}}$$

where *Z_o _*is the nozzle height above the horizontal disk plate, *V_i _*is the jet velocity at impingement and *V_j _*is the velocity at nozzle exit. It was maintained at 50 mm in this study. Furthermore, the jet diameter *D_i _*just before the impingement is calculated from the continuity equation just before impingement:

(15) $$D_{i}=D_{j}{\left(\frac{V_{j}}{V_{i}}\right)}^{0.5}$$

where *D_j _*is the nozzle diameter. The jet Reynolds number *Re_i _*based on the jet velocity and the diameter at the impingement point is given by:

(16) $$Re_{i}=\frac{\rho \times V_{i}\times D_{i}}{\mu }$$

It should be noted that the physical properties of water used in the above equations are estimated at the film temperature *T*_f_:

(17) $$T_{\text{f}}=\frac{T_{\text{j}}+T_{\text{wa}}}{2}$$

where *T*_j _is the temperature of the jet.

## Results and discussion

Uncertainty propagation technique is used to calculate how the uncertainties in each of the measured variables propagate into the value of the calculated quantities. The method for determining this uncertainty propagation is described in Taylor and Kuyatt [36]. The uncertainties in the measured quantities are considered as follows: in temperature, ± 0.4°C; in length and diameter, ± 0.05 mm; in nanofluid mass, ± 0.1 g; in electric heating power, ± 1.5% and in flow rate, ± 2%. The estimated uncertainity in the calculated Peclet and Nusselt numbers falls within ± 2% and ± 8.7%, respectively.

**Figure 6:**
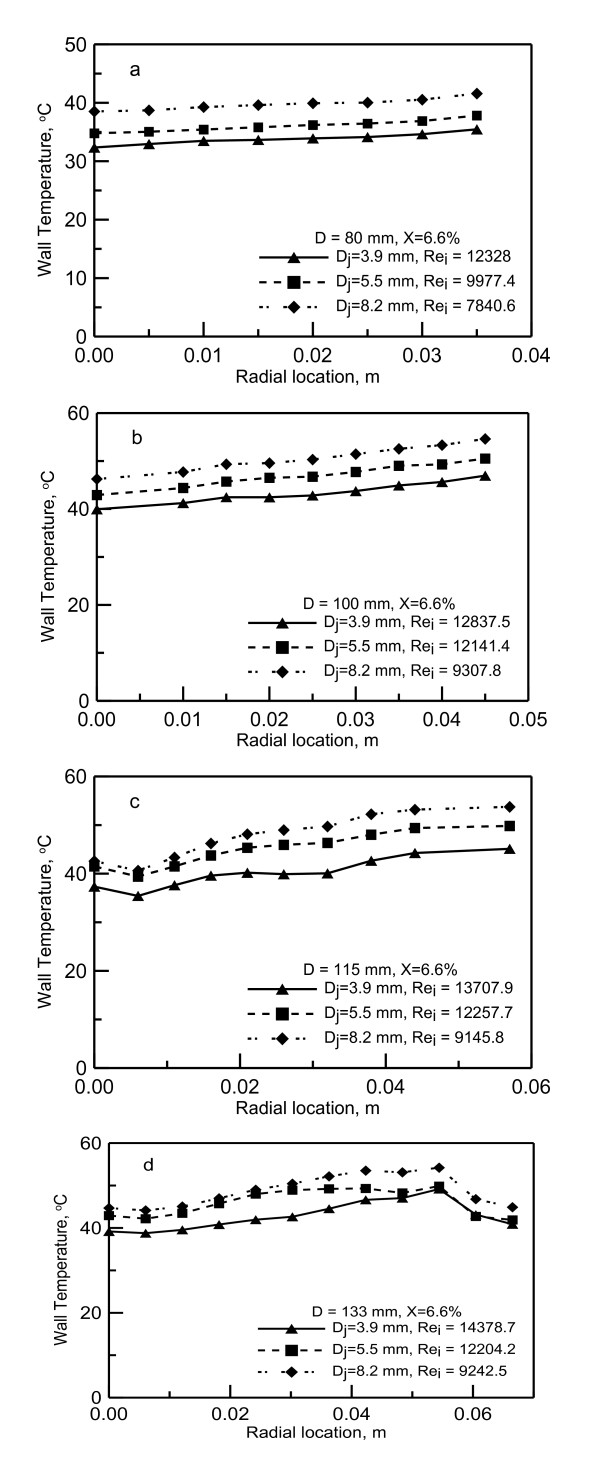
**Measured local wall temperature along the radial direction**. (**a**) Data for heating disk of diameter *D *= 0.08 m, (**b**) 0.10 m, (**c**) 0.115m and (**d**) 0.133 m.

Samples of the measured wall temperature along the radial direction are shown in Figure 6. As seen in this figure, the lowest wall temperature is observed at the impingement point (radial distance = 0). For the same Reynolds number, the wall temperature increases along the radial direction. This indicates that the heat transfer coefficient decreases along the radial direction. The reduction in the heat transfer is caused by the increase in the liquid film thickness and the decrease in the film velocity along the radial direction. As the jet Reynolds number increases, the wall temperature decreases as seen in Figure 6. These results reveal that the jet Reynolds number plays a major role in the heat transfer process.

**Figure 7:**
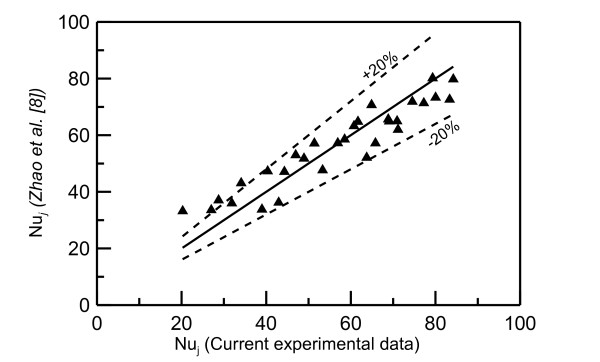
**Comparison with the model of Zhao and Masuoka **[8]. Black triangles represent current data of water jets.

**Figure 8:**
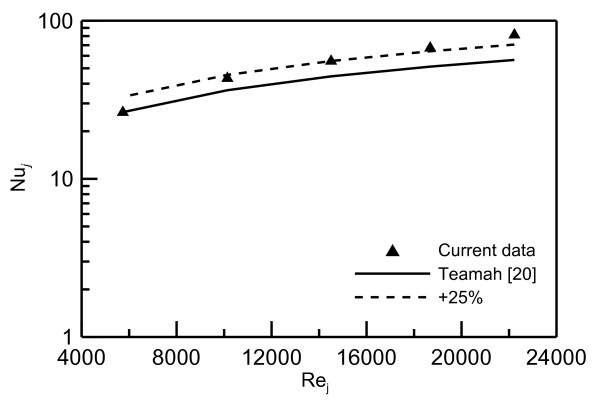
**Comparison with the model of Teamah and Farahat **[10].

Comparison between the current data for water jet and the model of Zhao et al. [8] (Equation 1) and Teamah and Farahat [10] (Equation 2) are shown in Figures 7 and 8, respectively. The current data fall within ± 20% of Zhao et al.'s model [8]; however, Teamah and Farahat's model [10] under-predicts the current data by about 25%. Nevertheless, these comparisons indicate that the current data fall within the predicted models.

**Figure 9:**
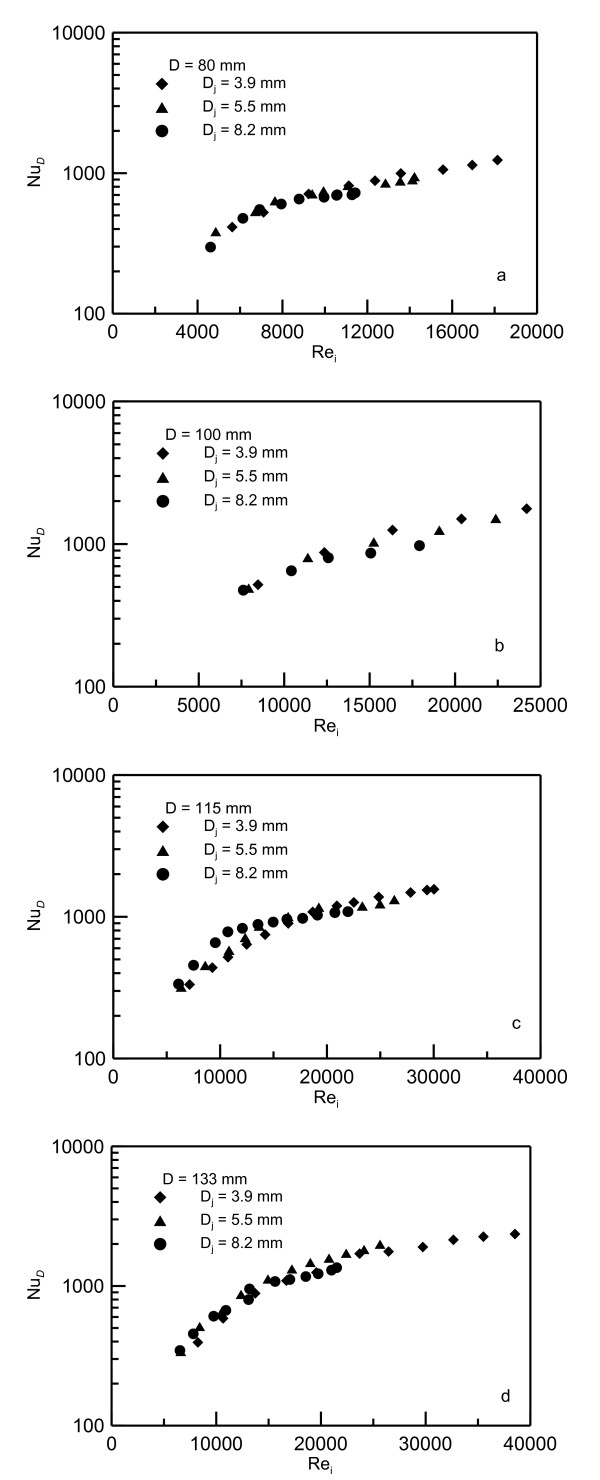
**Nusselt numbers versus Reynolds numbers for water**. (**a**) Data for heating disk of diameter *D *= 0.08 m, (**b**) 0.10 m, (**c**) 0.115m and (**d**) 0.133 m.

**Figure 10:**
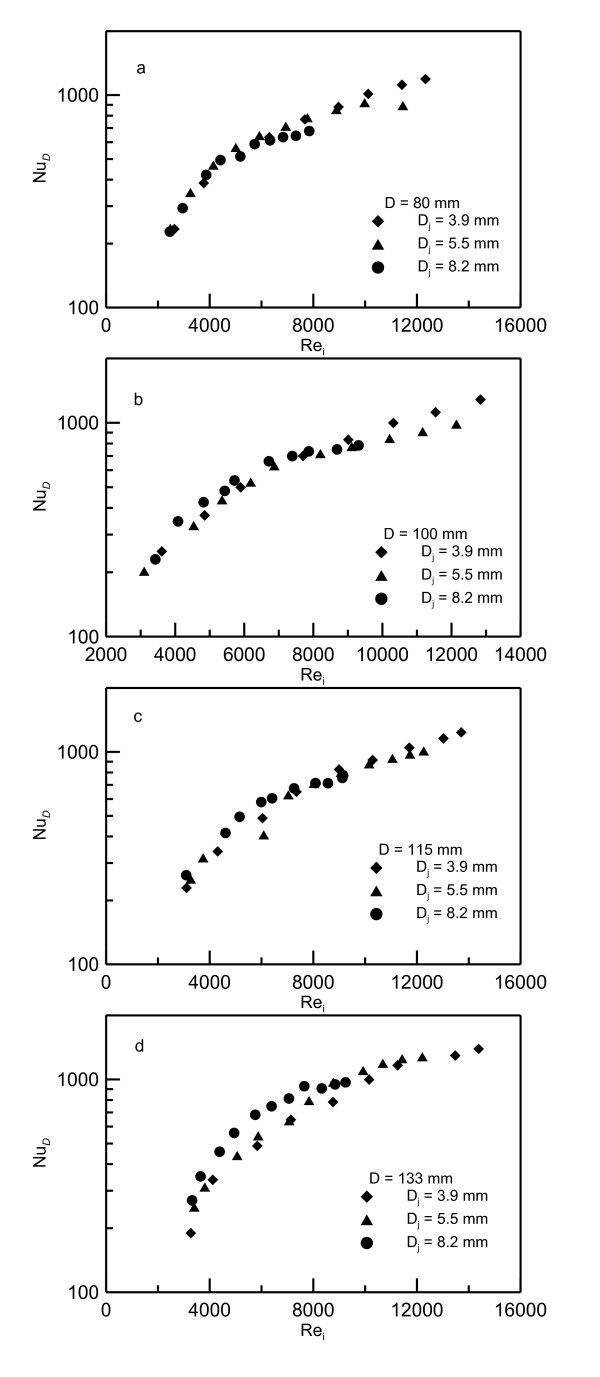
**Nusselt numbers versus Reynolds numbers for 6.6% nanofluid concentration**. (**a**) Data for heating disk of diameter *D *= 0.08 m, (**b**) 0.10 m, (**c**) 0.115m and (**d**) 0.133 m.

**Figure 11:**
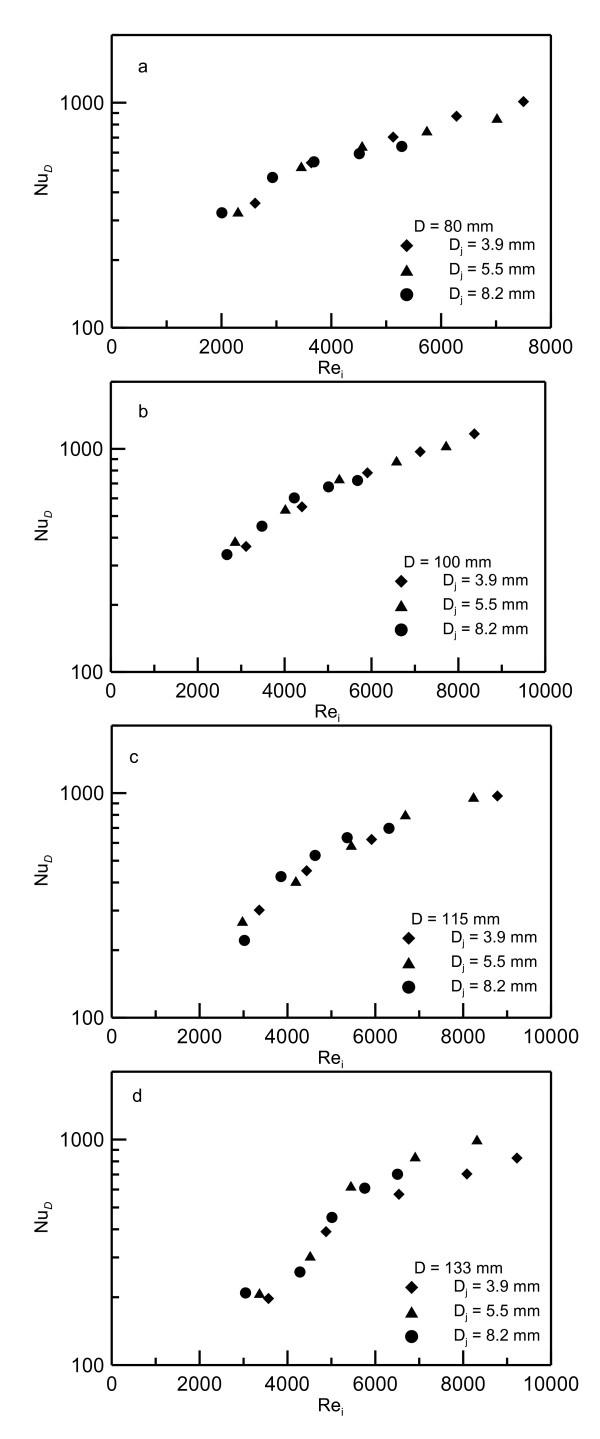
**Nusselt numbers versus Reynolds numbers for 10% nanofluid concentration**. (**a**) Data for heating disk of diameter *D *= 0.08 m, (**b**) 0.10 m, (**c**) 0.115m and (**d**) 0.133 m.

Preliminary investigation has shown that the best presentation of the data is to be in terms of the Nusselt number *Nu_D _*based on disk diameter versus the jet Reynolds number *Re_i _*based on jet velocity and diameter at the impingement point. It should be noted that using the *V_i _*at the impingement point in estimating the Reynolds number takes into account the effect of the nozzle height. Figures 9,10,11 show the data of the mean Nusselt numbers versus the Reynolds number *Re_i _*for the nanoparticle concentrations of 0%, 6.6% and 10%, respectively, for different jets *D_j _*= 0.0039, 0.0055 and 0.0082 m and heated disks of diameters *D *= 0.080, 0.100, 0.115 and 0.133 m. The general trend drawn from these figures is that as the Reynolds number increases the Nusselt number increases too. It should be noted that using Re_i _in the data presentation eliminates the effect of jet diameter and height *Z_o _*on *Nu_D _*profiles as shown in this figures. Figure 9a,b,c,d show that all the data for pure water are heating disk-independent and, therefore, collapse on one curve. As the nanoparticle concentration percentage increases, the absolute viscosity increases which leads to reduced Reynolds number as seen in Figure 10a,b,c,d and Figure 11a,b,c,d for 6.6% and 10% concentrations, respectively. In spite of using different jet diameters, the experimental data collapse on one curve for each heater disk. It was also found that presenting the data in terms of Reynolds number at the impingement disk can take into account the effect of nozzle height and jet diameter. In other words, using this choice of Reynolds number makes *Nu_D _*less sensitive to nozzle diameter change.

**Figure 12:**
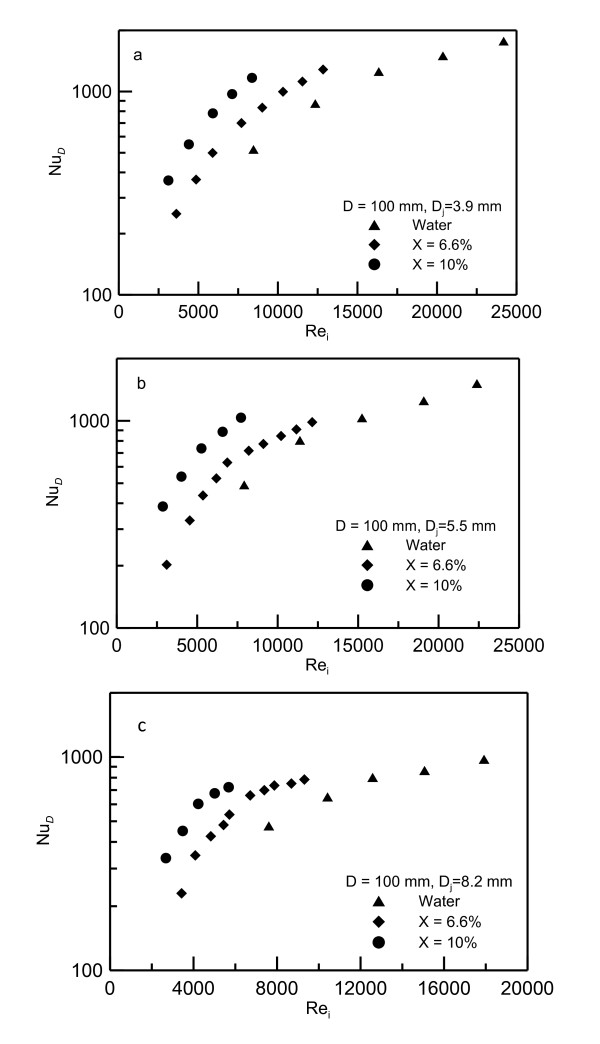
**Comparison between Nusselt numbers for water and nanofluid for 100-mm diameter heater**. (**a**) Cross plots for *D_j _*= 0.0039 m, (**b**) for *D_j _*= 0.0055 m and (**c**) for *D_j _*= 0.0082 m.

**Figure 13:**
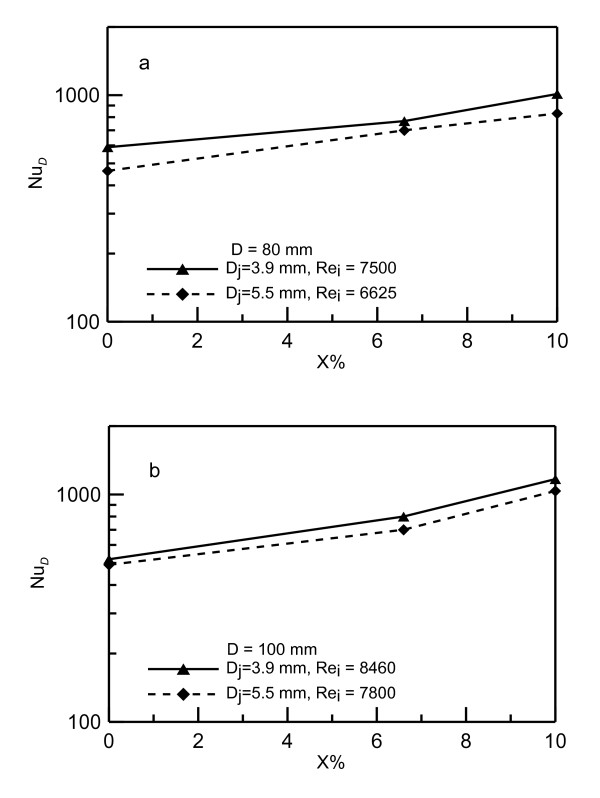
**Enhancement in Nusselt number due to an increase in the nanofluid concentration**. (**a**) Data for heating disk of diameter *D *= 0.08 m and (**b**) 0.10 m.

Cross plots of Figures 9 to 11 for fixed disk diameter *D *= 0.100 m and for different concentrations are shown in Figure 12a,b,c. Figure 12a shows the cross plot for *D_j _*= 0.0039 m, (b) for *D_j _*= 0.0055 m and (c) for 0.0082 m. Figure 12a,b,c shows increase in *Nu_D _*as the concentration increases. For example, at *Re_i _*= 8,000 the Nusselt number *Nu_D _*increases from 500 at zero concentration to 700 at 6.6% concentration up to 1,000 at 10% concentration. For the same Reynolds number, the experimental data show an increase in Nusselt number that can reach up to 100% at higher concentration. This result indicates that using nanofluid as a heat transfer carrier can enhance the heat transfer process. It should be noted that, as the concentration increases, Reynolds number decreases as mentioned earlier, and the data for 10% concentration is shifted to the left and that for pure water is on the right. It means that the increase in Nusselt number is on the account of force required to push the flow. Figure 13 is constructed to show the effect of nanofluid concentration on the Nusselt number. The data in this figure is extracted from Figure 12. As seen in Figure 13, the increase in Nusselt number can reach up to 40% and 75% at 6.6% and 10% concentrations, respectively.

**Figure 14:**
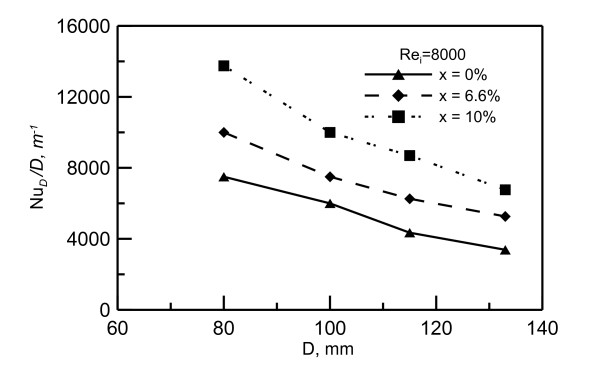
**The effect of disk diameter and nanofluid concentration on heat transfer**.

The effect of disk diameter and noanofluid concentration is shown in Figure 14. In this figure, the heat transfer coefficient is presented in terms of *Nu_D _*/*D *or (*h */*k*) versus disk diameter *D*. This ratio was chosen such that Nusselt number be independent of disk diameter *D*. These data indicate that heat transfer coefficient decreases as heating disk diameter increases. This decrease could be attributed to the fact that, as the liquid film thickness increases along the radial direction, the film velocity decreases. Therefore, as the heating disk diameter increases, the local heat transfer coefficient along the radial direction of the disk decreases. Consequently, the average heat transfer coefficient decreases. Furthermore, the opportunity of the flow to turn from shooting before the hydraulic jump to a streaming flow after the hydraulic jump increases as the disk diameter increases. The sudden increase in the film thickness after the hydraulic jump leads to a significant decrease in the heat transfer coefficient. The effect of nanofluid concentration on the heat transfer is also shown in Figure 14. This figure shows that increasing the concentration enhances the heat transfer coefficient. Using 10% concentration can increase the heat transfer (*Nu_D _*/*D *or *h */*k*) about 60%. Moreover, the actual increase in the heat transfer coefficient should be more due to the increase in thermal conductivity k of the nanofluid.

**Figure 15:**
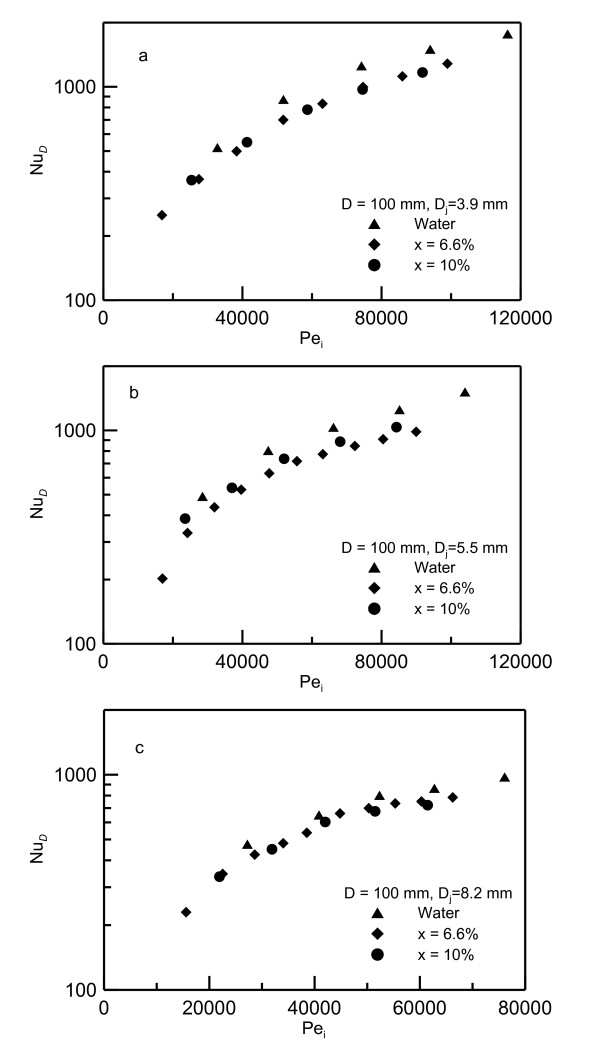
**Nusselt numbers versus Peclet numbers for 100 mm diameter heater**. (**a**) Data for jet diameter D_j _= 0.0039 m, (**b**) 0.0055 m and (**c**) 0.0082 m.

Figure 15a,b,c presents the profiles of Nusselt number versus Peclet number, *Pe_i_*, for disk diameter *D *= 0.100 m and for different concentrations. Figure 15a shows the data for jet diameter *D_j _*= 0.0039 m, (b) for 0.0055 m and (c) for 0.0082 m, respectively. As shown in this figure, the data of various concentrations are collapsed on one curve when *Pe_i _*is used in presenting the data. The nanoparticle concentration has a strong impact on viscosity, and consequently, on Prandtl number. Therefore, as the nanoparticle concentration increases from 05 to 10%; the viscosity increases about 150%. However, using Peclet number in Figure 15a,b,c ensures the effect of Prandtl number which makes the data to collapse on one curve.

**Figure 16:**
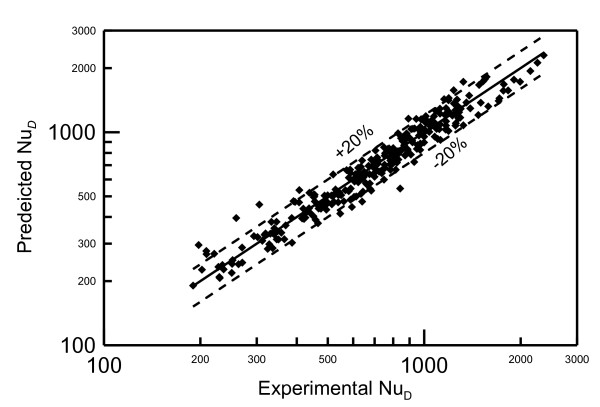
**Comparison between the predicted and the experimental Nusselt numbers**. Black squares represent predicted Nusselt number using Equation 18 at experimental conditions of Nusselt number shown on x-axis.

Different correlations were examined to fit the data in Figure 15a,b,c. It is found that the best correlation trend is obtained by correlating the experimental data in terms of Pe_i_, nanofluid concentration represented by (1-*X*) where *X *is the percentage of nanoparticle concentration, and heating disk to jet diameter ratio *D */*D_i_*. The effect of diameter ratio is chosen to be similar to that of Zhao et al.'s model [8] in Equation 1. The correlation obtained is:

(18) $$Nu_{D}\;=\;\left(0.2{\left(\frac{D}{D_{i}}\right)}^{-0.5}\;-5.022{\left(\frac{D}{D_{i}}\right)}^{-2}\right){\left(1-X\right)}^{2.136}P{e_{i}}^{0.933}$$

where 0 ≤ × ≤ 0.1 with a correlation coefficient *R *= 96.5%. Comparison between the predictions of this correlation and the experimental data are shown in Figure 16. This figure shows that 96% of the data fall within error bands of ± 20%.

## Conclusions

Experimental investigation to study the heat transfer between a vertical round water jet having nanoparticles of aluminum oxide, and a horizontal circular round surface is carried out for different jet flow rates, jet diameters, nanoparticle concentrations and heating surface diameters. Three different nanoparticle concentrations 0%, 6.6% and 10% are used in the current investigation. For the same Reynolds number, the experimental data show an increase in the Nusselt number that can reach up to 100% for some higher concentrations. This result indicates that using nanofluid as a heat transfer carrier can enhance the heat transfer process. It was also found that presenting the data in terms of Reynolds number at impingement jet diameter can take into account the effect of jet heights and nozzle diameters. The data have also indicated that increasing heating disk diameter decreases the heat transfer coefficient. Experimental data was correlated in terms of jet Peclet number *Pe_i_*, nanofluid concentration presented by (1-*X*) and diameter ratio (*D */*D_i_*) with a correlation coefficient of *R *= 96.5%. Predictions of the obtained correlation fall within error bands of ± 20% of the experimental data.

## Competing interests

The authors declare that they have no competing interests.

## Authors' contributions

OZ designed and installed experimental rig, carried out experimental runs, analyzed experimental data and wrote and prepared manuscript. MA participated in installing experimental rig, data analysis, preparing and writing the manuscript. All authors read and approved the final manuscript.

## Appendix

Nomenclature

*A *Area of disk, πD^2^/4, square meter

*A_k _*Area element assigned to each thermocouple, square meter

*A_j _*Area of jet nozzle, πD_j_^2^/4, square meter

*C *Specific heat, joules per kilogram Kelvin

*D *Disk diameter, meter

*D_i _*Jet diameter at impingement, meter

*D_j _*Nozzle diameter, meter

*h *Average heat transfer coefficient, watts per square meter Kelvin

*k *Thermal conductivity, watts per meter Kelvin

*Nu_D _*Nusselt number, *hD */*k*

*Nu_j _*Nusselt number, *hD_j _*/*k*

*Pe_i _*Peclet number, *Re_i _Pr*

*Re_i _*Jet Reynolds number at impingement, *ρV_i _D_i _*/*μ*

*Re_j _*Nozzle Reynolds number, *ρV_j _D_j _/μ*

*T *Temperature, Kelvin

*V_i _*Jet velocity at impingement, meter per second

*V_j _*Velocity at nozzle exit, meter per second

*Q *Heat transfer, watt

*q *Heat flux, *Q */*A*, watt per square meter

*X *Nanofluid particle mass concentration, percent

*Z_o _*Height of jet above surface disk, meter

Greek symbols

*ν *kinematic viscosity, square meter per second

*μ *viscosity, Pascal second

*ρ *fluid density, kilogram per cubic meter

*φ *volumetric concentration, percent

Subscripts

a average

b base fluid

ele electrical

i impingement

ins insulation

j jet

k local temperature

loss losses

m metal

p nanoparticle

w wall
